# Targeted co-delivery of curcumin and erlotinib by MoS_2_ nanosheets for the combination of synergetic chemotherapy and photothermal therapy of lung cancer

**DOI:** 10.1186/s12951-023-02099-4

**Published:** 2023-09-16

**Authors:** Zhihuai Chen, Xinqi Wei, Yunru Zheng, Zongwei Zhang, Wang Gu, Wenjun Liao, Hua Zhang, Xiaoying Wang, Jian Liu, Hua Li, Wei Xu

**Affiliations:** 1 Institute of Structural Pharmacology & TCM Chemical Biology, College of Pharmacy, Fujian University of Traditional Chinese Medicine, No. 1, Qiuyang Road, Fuzhou, 350122 Fujian China; 2 Key Laboratory of Gastrointestinal Cancer, Ministry of Education, School of Basic Medical Sciences, Fujian Medical University, Fuzhou, 350005 China

**Keywords:** MoS_2_ nanosheets, Biotin, NIR light-responsive drug release, Synergistic chemotherapy, Photothermal therapy

## Abstract

**Background:**

Curcumin (Cur), a bioactive component of Chinese traditional medicine, has demonstrated inhibitory properties against cancer cell proliferation while synergistically enhancing the anticancer efficacy of erlotinib (Er). However, the individual limitations of both drugs, including poor aqueous solubility, lack of targeting ability, short half-life, etc., and their distinct pharmacokinetic profiles mitigate or eliminate their combined antitumor potential.

**Results:**

In this study, we developed a molybdenum disulfide (MoS_2_)-based delivery system, functionalized with polyethylene glycol (PEG) and biotin, and co-loaded with Cur and Er, to achieve efficient cancer therapy. The MoS_2_-PEG-Biotin-Cur/Er system effectively converted near-infrared (NIR) light into heat, thereby inducing direct photothermal ablation of cancer cells and promoting controlled release of Cur and Er. Biotin-mediated tumor targeting facilitated the selective accumulation of MoS_2_-PEG-Biotin-Cur/Er at the tumor site, thus enhancing the synergistic antitumor effects of Cur and Er. Remarkably, MoS_2_-PEG-Biotin-Cur/Er achieved the combination of synergistic chemotherapy and photothermal therapy (PTT) upon NIR irradiation, effectively suppressing lung cancer cell proliferation and inhabiting tumor growth in vivo.

**Conclusions:**

The as-synthesized MoS_2_-PEG-Biotin-Cur/Er, featuring high targeting ability, NIR light-responsive drug release, and the integration of synergistic chemotherapy and PTT, may provide a promising strategy for the treatment of lung cancer in clinical practice.

**Graphical Abstract:**

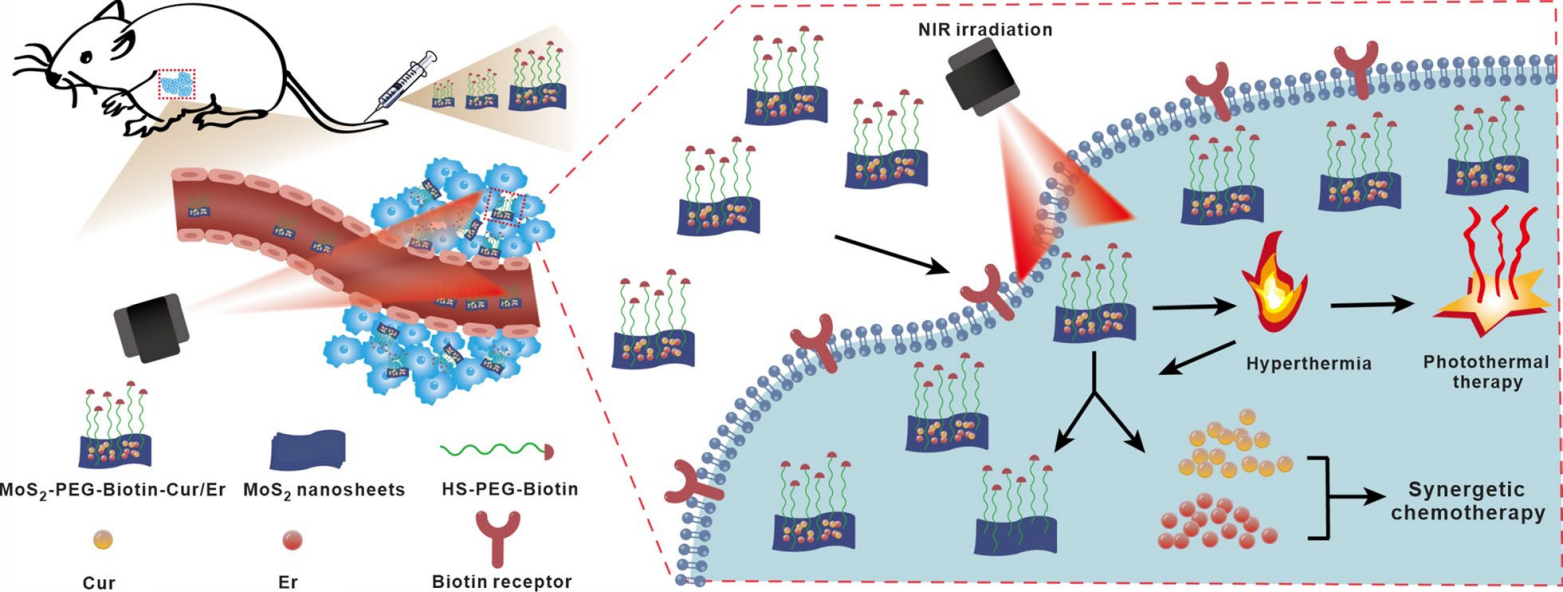

**Supplementary Information:**

The online version contains supplementary material available at 10.1186/s12951-023-02099-4.

## Introduction

Lung cancer represents a significant public health concern, with devastating global impacts of up to 2.2 million new cases and 1.8 million deaths in 2020, surpassing all other cancers [1, 2]. Epidermal growth factor receptor (EGFR) is highly expressed in lung cancer patients and plays a crucial role in various malignancy-related processes [3, 4]. Although several EGFR tyrosine kinase inhibitors (EGFR-TKIs), such as erlotinib (Er), have been developed, acquired resistance to these therapies is a major challenge, limiting their long-term effectiveness for lung cancer [5, 6].

To overcome this obstacle, researchers have combined Er with curcumin (Cur), an active ingredient in traditional Chinese medicine, to enhance the therapeutic effect against lung cancer [7]. The combination of Cur and Er has demonstrated low dose administration, high efficacy, minimal side effects, and the ability to prevent or reverse multidrug resistance [8–11]. Several studies suggest that Cur can inhibit Er resistance by maintaining ikappa-B expression levels and downregulating PI3K expression in the EGFR downstream signaling pathway, thereby promoting the release of apoptotic proteins caspase-3 and caspase-9 [12, 13]. However, challenges remain in the clinical translation of this combination therapy, including poor water solubility, a short half-life, non-targeting, and pharmacokinetic hindrances [14, 15]. Therefore, designing appropriate drug delivery systems for Cur and Er is essential. While numerous Cur- or Er-carrying nano-delivery systems have been developed in recent years [16, 17], co-loaded systems for Cur and Er are rare.

Molybdenum disulfide (MoS_2_), a widely used two-dimensional material comprised of three atomic layers of centrosymmetric S-Mo-S, has been widely investigated for applications in electronic devices and catalysis because of its unique properties [18]. In biomedical fields, MoS_2_ exhibits good biocompatibility, abundant chemical reaction sites, and strong absorption in the near infrared region, making it a photothermal material for biological applications [19, 20]. It’s noteworthy that monolayer MoS_2_ has a direct bandgap of 1.8 eV, indicating a high photothermal conversion efficiency [21]. Owing to its ultrathin thickness and large surface area, MoS_2_ possess a larger specific surface area and higher drug loading capacity than some inorganic photothermally active nanomaterials, facilitating the delivery of drugs [22–24]. In this study, Liu et al. successfully utilized PEG-modified MoS_2_ nanosheets as carriers to load several anticancer drugs and demonstrated that MoS_2_ outperforms other common nanomaterials, such as graphene oxide, in terms of drug-loading capacity [25]. Further, we have developed a series of MoS_2_-based nano-delivery systems targeted for multiple medicines and the contrast agent gadolinium, showcasing the capability of MoS_2_ as a safe and efficient drug carrier capable of loading, delivering, and controlling the release of drugs, contributing to the development of a Cur and Er co-loaded delivery system to achieve synergistic chemotherapy of lung cancer [26–28]. To enhance cell selectivity, we intend to functionalize some tumor-targeting ligands on the surface of MoS_2_ nanosheets, such as biotin. Biotin, also known as vitamin H, is an essential micronutrient for maintaining natural growth, development, and normal physiological functions in humans [29]. Compared to normal cells, cancer cells require additional biotin to support rapid growth and proliferation, resulting in overexpression of biotin receptors in various cancer cells, such as A549 cells (lung cancer) [30, 31]. Biotin-functionalized nanodrug delivery systems have been extensively developed, which selectively deliver loaded drugs to tumor cells, demonstrating efficient targeting ability [31–34].

In this study, a multifunctional MoS_2_-based nanoplatform, functionalized with biotin, was devised, capable of targeted delivery of Cur and Er and controlled drug release, thereby achieving synergetic chemotherapy and photothermal therapy for lung cancer. The resultant MoS_2_-PEG-Biotin-Cur/Er complex exhibited excellent antitumor efficacy, as confirmed by in vitro and in vivo experiments.

## Experimental section

### Materials

MoS_2_ crystal was purchased from Muke Nano Science and Technology Co., Ltd. (Nanjing, China). HS-PEG-NH_2_ (molecular weight (MW): 5000) was provided by Xi'an Ruixi biological Co., Ltd. (Xi'an, China). 3-[4,5-Dimethylthiazol-2-yl]-2,5-diphenyl tetrazolium bromide (MTT), Hoechst 33,258, and Ham’s F12K medium were obtained from Sigma-Aldrich (St. Louis, USA). Trypsin-ethylenediaminetetraacetic acid (EDTA) and fetal bovine serum (FBS) were purchased from Gibco-BRL (Burlington, Canada). An Annexin V-APC/7-AAD apoptosis detection kit was supplied by Biolegend Company (San Diego, USA). All other reagents were purchased from J&K Scientific, Ltd., and used without further purification.

### Synthesis of HS-PEG-Biotin

Biotin (50 mg) was dissolved in dimethyl sulfoxide (DMSO) prior to the addition of EDC (17 mg) and NHS (9 mg). After 2 h of reaction, the activated biotin was added dropwise to a 40 mg/mL HS-PEG-NH_2_ aqueous solution (5 mL) in 5 min, followed by 24 h of vigorously agitating. After centrifugation, the resulting solution was dialyzed (3.5 kDa) against deionized water for two days, and HS-PEG-Biotin was obtained by lyophilization.

### Preparation of MoS_2_-PEG-Biotin

Single-layered MoS_2_ nanosheets were synthesized according to the previously reported method [25]. To prepare MoS_2_-PEG-Biotin, SH-PEG-Biotin was grafted on the surface of MoS_2_ via binding thiolated molecules to the defect sites of the nanosheets. Briefly, 10 mg of HS-PEG-Biotin was added to 2 mL of an aqueous solution containing MoS_2_ nanosheets (1 mg/mL). After sonication for 10 min and stirring for 24 h, the mixed solution was dialyzed (100 kDa) against deionized water for a week to remove excess HS-PEG-Biotin, and the resulting MoS_2_-PEG-Biotin was stored at 4 °C for further use.

### Characterization

Fourier transform infrared (FT-IR) spectra were obtained using a Tensor 27 FT-IR spectrometer (Bruker), while UV–Vis–NIR absorption spectra were obtained using a UV-2700 spectrophotometer (Shimadzu). The morphologies of the MoS_2_ and MoS_2_-PEG-Biotin nanosheets were analyzed by atomic force microscopy (AFM, Bruker). Temperature curves were acquired using an infrared thermal camera (220 s, Fotric AnalyzIR).

### Hemolysis assay

To determine the hemolytic effects of MoS_2_-PEG-Biotin, red blood cells (0.8 mL) from a healthy mouse were incubated with varying concentrations of MoS_2_-PEG-Biotin dispersions (0.2 mL) for 2 h at high speed before being centrifuged. The absorbance of the supernatant was measured using a UV–Vis spectrophotometer, with negative and positive controls consisting of PBS and deionized water, respectively. The hemolysis percentage was calculated as follows: hemolysis percent (%) = (A _treated_–A _negative_)/(A _positive_–A _negative_) × 100%, where A represents absorbance.

### Cytotoxicity of MoS_2_-PEG-Biotin

The cytotoxicity of MoS_2_-PEG-Biotin was assessed using the MTT assay. A549 or HELF cells were seeded into 96-well plates at densities of 4 × 10^3^ and 5 × 10^3^ cells/well for 24 h, respectively, followed by incubation with cell medium containing gradient concentrations of MoS_2_-PEG-Biotin for another 24, 48, and 72 h. Afterward, the cells were washed with PBS twice and treated with the MTT agent (100 μL) for 4 h before analysis using a microplate reader.

### Drug loading and release

Cur and Er were loaded onto MoS_2_-PEG-Biotin nanosheets by stirring Cur (0.4 mg/mL) in DMSO and MoS_2_-PEG-Biotin aqueous dispersion (0.4 mg/mL), followed by the addition of Er solution (0.2 mg/mL) to achieve a final concentration of 70% DMSO. After centrifugation to remove undissolved drug solids and ultrafiltration to eliminate solubilized drugs, the amount of Cur and Er loaded onto MoS_2_-PEG-Biotin-Cur/Er was determined by measuring absorption at 430 nm and 335 nm after subtraction of the contribution from MoS_2_-PEG-Biotin, respectively.

Release behavior was evaluated by immersing MoS_2_-PEG-Biotin-Cur/Er nanosheets in a dialysis bag in PBS with 0.5 wt% tween-80 and exposing them to an 808 nm laser at different densities for 10 min. Dialysis solution (1 mL) was collected at predetermined time points, measured using UV spectrophotometry, and poured back into the system to maintain a constant volume. The amount of Cur and Er released was determined by UV absorption peaks at 430 nm and 335 nm, respectively.

### Cellular uptake

Fluorescent labeling of MoS_2_-based nanosheets was achieved via physical adsorption of rhodamine B (RB) onto MoS_2_-PEG-Biotin by mixing RB aqueous solution (1 mg/mL) with MoS_2_-PEG-Biotin suspension (0.5 mg/mL), followed by ultrafiltration to remove unbonded RB. The labeled MoS_2_-PEG-biotin-RB was preserved at 4 °C for subsequent use.

Cellular uptake of biotin-modified MoS_2_ nanosheets was investigated by exposing A549 cells (biotin receptor positive) and HELF cells (biotin receptor negative) to MoS_2_-PEG-RB or MoS_2_-PEG-Biotin-RB ([RB] = 20 μg/mL) for 2 h, washing with PBS, and analyzing fluorescence intensity by confocal microscopy. Additionally, fluorescence intensity of the cells was measured using a flow cytometer after three washes with PBS, harvesting with trypsin, and resuspending in PBS.

### In vitro combination therapy of MoS_2_-PEG-Biotin-Cur/Er

The in vitro cytotoxicity of MoS_2_-PEG-Biotin-Cur/Er was evaluated in A549 cells using the MTT assay. Cells were incubated with Cur, Er, Cur + Er, and MoS_2_-PEG-Biotin-Cur/Er at a range of concentrations for 2 h, washed with PBS, and assessed for viability after 48 h of treatment with fresh medium.

For combination therapy, A549 cells were divided into six groups: a control group, Cur, Er, Cur + Er, MoS_2_-PEG-Biotin, and MoS_2_-PEG-Biotin-Cur/Er ([MoS_2_-PEG-Biotin] = 100 μg/mL, [Cur] = 20 μg/mL, and [Er] = 10 μg/mL). After 2 h of drug exposure, cells were washed twice with PBS and provided with fresh medium. The NIR irradiation groups were exposed to an 808 nm laser (1 W/cm^2^) for 10 min, while others served as controls. After treatment, all cells were incubated for another 48 h before the MTT assay was performed to measure cell viability.

### Cell apoptosis

To further assess the therapeutic efficacy in vitro, cell apoptosis was analyzed using flow cytometry with an Annexin V-FITC/PI apoptosis detection kit. A549 cells were pre-seeded in 6-well plates and divided into eight groups: (1) Cell medium as a control, (2) Cur, (3) Er, (4) Cur + Er, (5) MoS_2_-PEG-Biotin, (6) MoS_2_-PEG-Biotin + NIR, (7) MoS_2_-PEG-Biotin-Cur/Er, and (8) MoS_2_-PEG-Biotin-Cur/Er + NIR ([MoS_2_-PEG-Biotin] = 100 μg/mL, [Cur] = 20 μg/mL, and [Er] = 10 μg/mL). After 2 h of drug treatment, the cells were washed with PBS and given fresh medium, and the wells of the NIR irradiation groups were exposed to an 808 nm laser (1 W/cm^2^) for 10 min. After another 48 h of incubation, Annexin V-FITC/PI staining was performed to analyze cell apoptosis.

### Tissue biodistribution

The tissue biodistribution of MoS_2_-PEG-Biotin-Cur/Er was investigated in lung cancer cell-bearing nude mice. A549 cells (5 × 10^6^ cells) suspended in PBS (100 μL) were subcutaneously injected into the forelimbs of female BALB/C nude mice. Once the mean tumor volume reached approximately 200 mm^3^, the mice were randomly divided into two groups (n = 12 per group) and intravenously administered MoS_2_-PEG-Cur/Er or MoS_2_-PEG-Biotin-Cur/Er ([MoS_2_-PEG-Biotin] = 8 mg/kg, [Cur] = 1.6 mg/kg, and [Er] = 0.8 mg/kg). At predetermined time intervals (2, 6, 12, and 24 h), three random mice were sacrificed, and tissue samples (heart, liver, spleen, lung, kidney, and tumor) were collected, weighed, and digested by aqua regia. The amount of Mo in the tissues was then determined using inductively coupled plasma-mass spectrometry (ICP-MS).

### In vivo combination therapy of MoS_2_-PEG-Biotin-Cur/Er

In vivo combination therapy of MoS_2_-PEG-Biotin-Cur/Er was carried out using the tumor model established as described in the “Tissue biodistribution” section. When the mean tumor volume reached approximately 60 mm^3^, nude mice were randomly separated into eight groups (n = 5 mice/group) and injected with 200 μL of (1) PBS containing 0.5% DMSO (v/v) as a control, (2) MoS_2_-PEG-Biotin, (3) Cur, (4) Er, (5) Cur + Er, (6) MoS_2_-PEG-Biotin + NIR, (7) MoS_2_-PEG-Biotin-Cur/Er, and (8) MoS_2_-PEG-Biotin-Cur/Er + NIR ([MoS_2_-PEG-Biotin] = 8 mg/kg, [Cur] = 1.6 mg/kg, and [Er] = 0.8 mg/kg). After 12 h of intravenous injection, nude mice in groups (1), (3), (4), (5), (6), and (8) were treated with NIR irradiation (1 W/cm^2^) for 10 min, while real-time temperature changes of the tumors were monitored using a FLIR thermal camera. The weight and tumor size of each mouse were recorded every three days. The tumor volume was calculated using the formula: V (volume, mm^3^) = length (mm) × width^2^ (mm^2^)/2. At 21 days post-treatment, all mice were sacrificed, and major organs were collected for hematoxylin–eosin (H&E) staining.

### Statistical analysis

Quantitative data are presented as the mean ± SD of at least three independent experiments. Statistical significance was assessed using Student’s *t*-test or one-way ANOVA with GraphPad Prism 5 software. A p-value less than 0.05 was considered statistically significant.

## Results and discussion

### Preparation and characterization of MoS_2_-PEG-Biotin

**Fig. 1:**
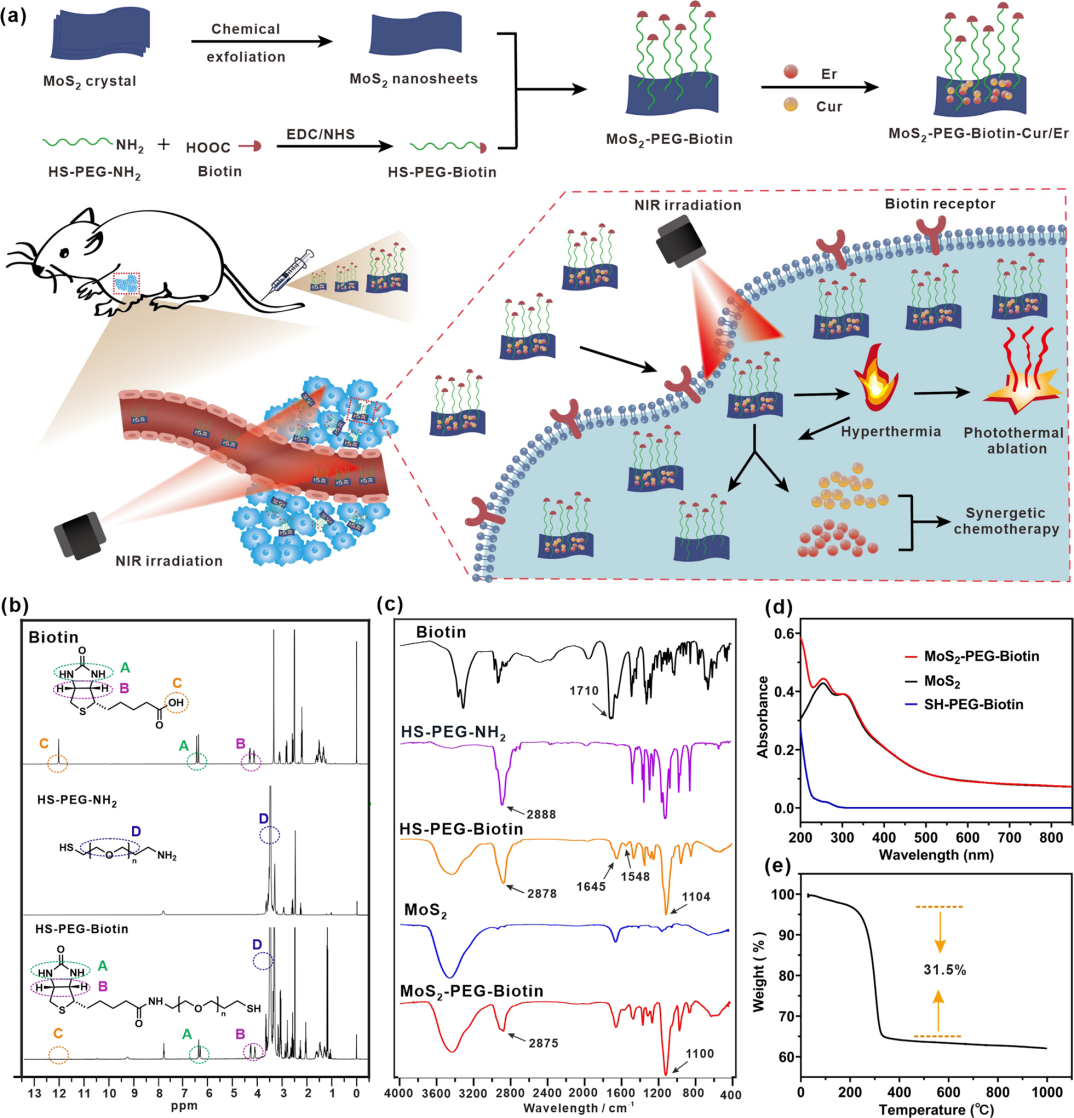
**a** A schematic of MoS_2_-PEG-Biotin as a carrier capable of achieving the targeted delivery of Cur and Er for the combination of synergetic chemotherapy and PTT. **b** ^1^H NMR spectra of biotin, HS-PEG-NH_2_, and HS-PEG-Biotin. **c** FT-IR spectra of biotin, HS-PEG-NH_2_, HS-PEG-Biotin, MoS_2_, and MoS_2_-PEG-Biotin. **d** UV–Vis–NIR spectra of MoS_2_ before and after HS-PEG-Biotin. **e** The weight loss curve of MoS_2_-PEG-Biotin

Figure 1a illustrates the preparation process of MoS_2_ nanosheets, which were modified with biotin to create a versatile carrier for the targeted co-delivery of Cur and Er, achieving a combined effect between synergistic chemotherapy and PTT. Biotin was first conjugated to PEG via the EDC/NHS technique, and the resulting HS-PEG-Biotin was verified by ^1^H NMR. Characteristic proton peaks of biotin and HS-PEG-NH_2_ segments were observed in the ^1^H NMR spectrum of HS-PEG-Biotin (Fig. 1b), such as at 6.52 and 6.36 ppm, and at 3.51 ppm for the secondary amine (–NH–) of biotin and methylene (–CH_2_–) of HS-PEG-NH_2_, respectively. The absence of the proton peak at 12.00 ppm of carboxyl (–COOH) group of biotin in the ^1^H NMR spectrum of HS-PEG-Biotin indicated the conjugation of HS-PEG-NH_2_ with biotin through the amido bond. To further confirm this, the FT-IR data of HS-PEG-Biotin was obtained using a Tensor 27 FT-IR spectrometer. As shown in Fig. 1c, in comparison with HS-PEG-NH_2_, HS-PEG-Biotin showed some additional absorption peaks at 1645 and 1548 cm^−1^ corresponding to the stretching vibration of amide I (C = O) and amide II (N–H), respectively, further confirming the successful preparation of HS-PEG-Biotin by the formation of the amido bond. For the modification of MoS_2_ with biotin, the thiol on the PEG terminal in the as-prepared HS-PEG-Biotin could be grafted onto the surface of MoS_2_ nanosheets by a simple mixing process [35]. After dialysis to remove impurities, FT-IR spectroscopy of MoS_2_-PEG-Biotin revealed some absorption bands at ~ 2875 and ~ 1110 cm^−1^ attributed to the C–H and C–O vibrations in HS-PEG-Biotin, confirming the presence of HS-PEG-Biotin on MoS_2_ nanosheets. MoS_2_-PEG-Biotin displayed a strong absorbance in the 200–250 nm wavelength range due to the grafted HS-PEG-Biotin, which was significantly higher than that of unmodified MoS_2_ (Fig. 1d). The weight percentage of HS-PEG-Biotin in MoS_2_-PEG-Biotin was determined to be ~ 31.5% through thermogravimetric analysis (Fig. 1e).

**Fig. 2:**
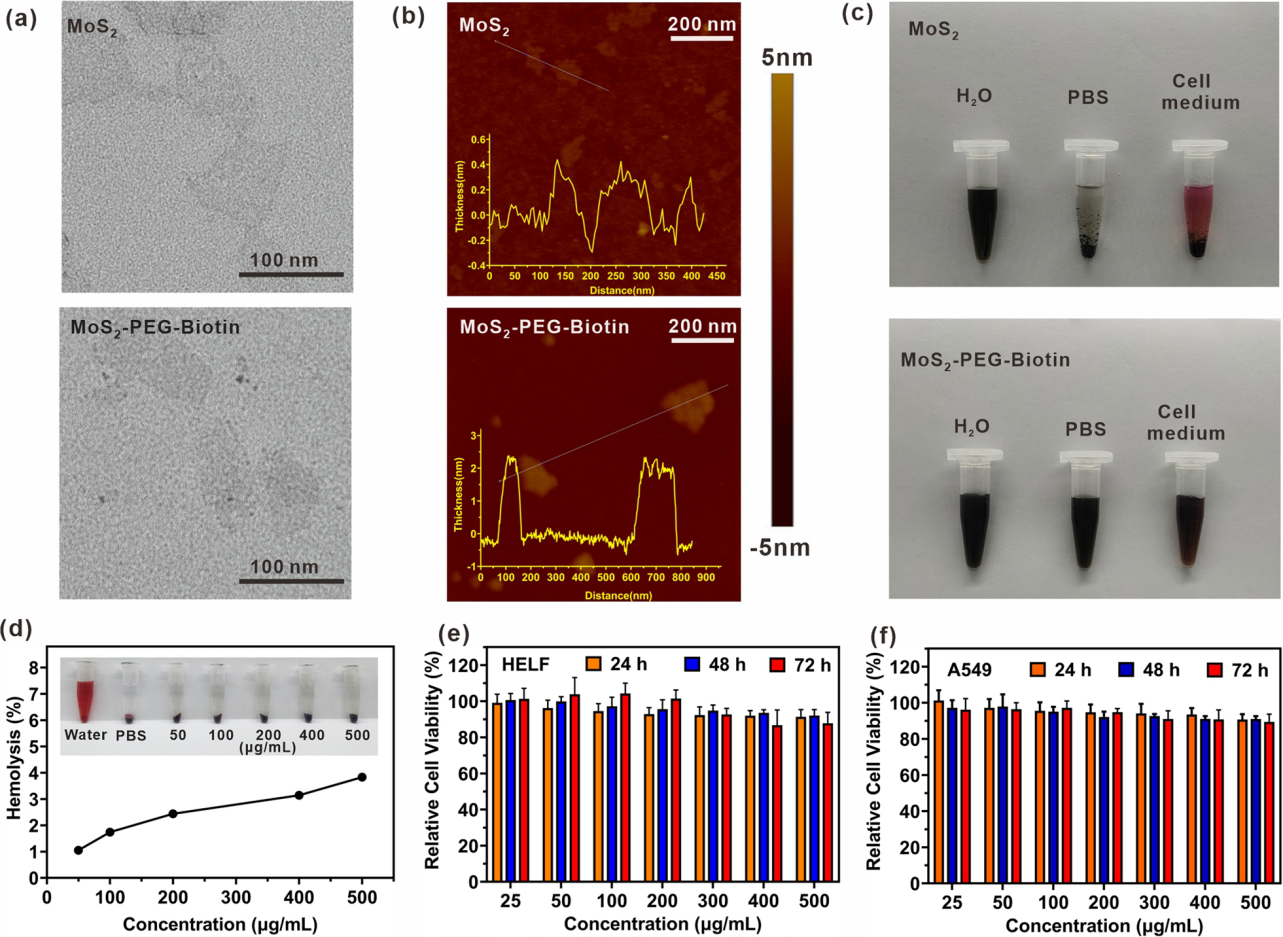
TEM (**a**) and AFM (**b**) images of MoS_2_ and MoS_2_-PEG-Biotin. **c** Stability of MoS_2_ and MoS_2_-PEG-Biotin in water, PBS, and cell medium within 7 days. **d** Hemolysis percentages of red blood cell survival by MoS_2_-PEG-Biotin. Inset: images of the direct observation of hemolysis. Relative cell viability of HELF (**e**) and A549 (**f**) cells incubated with various concentrations of MoS_2_-PEG-Biotin for 24, 48, and 72 h

The morphology and size-associated properties of MoS_2_ nanosheets before and after HS-PEG-Biotin loading were investigated using TEM, AFM, and DLS. MoS_2_-PEG-Biotin had a sheet-like morphology with an average size of ~ 120 nm (Fig. 2a), similar to single-layer MoS_2_ nanosheets (Additional file 1: Fig. S1). However, the thickness of MoS_2_ nanosheets increased from ∼ 0.7 nm to ∼ 2.3 nm after PEGylation, which indicated successful grafting of HS-PEG-Biotin on the surface of MoS_2_ nanosheets (Fig. 2b).

Having confirmed the successful synthesis of MoS_2_-PEG-Biotin, we assessed the carrier's physiological stability. As demonstrated in Fig. 2c, MoS_2_-PEG-Biotin was well-dispersed in DI water, PBS, and cell medium for a week. In contrast, MoS_2_ nanosheets rapidly aggregated in PBS and cell medium, due to the screening of the electrostatic charge on the surface [36]. The high physiological stability provided by PEG is essential for the application of MoS_2_-PEG-Biotin in biological fields.

### Biocompatibility of MoS_2_-PEG-Biotin

The biocompatibility of MoS_2_-PEG-Biotin was evaluated prior to its intravenous administration. After incubation with RBCs and centrifugation, the supernatants of MoS_2_-PEG-Biotin solutions at concentrations ranging from 50 to 500 μg/mL were clear and transparent, with negligible hemolysis (Fig. 2d). Meanwhile, the hemolysis ratio of the samples was calculated by measuring the absorbance of the supernatants at a wavelength of 541 nm. All the hemolysis percentages of the samples in group (3) were below 4%, indicating the good blood compatibility of MoS_2_-PEG-Biotin. Cytotoxicity of MoS_2_-PEG-Biotin was assessed using the MTT assay. As shown in Fig. 2e and f, over 90% of HELF and A549 cells remained viable even when incubated with MoS_2_-PEG-Biotin at concentrations up to 500 μg/mL for 72 h, suggesting low cytotoxicity. Such excellent blood compatibility and low cytotoxicity are expected to advance the applications of MoS_2_-PEG-Biotin in biological fields.

### Photothermal performance of MoS_2_-PEG-Biotin

**Fig. 3:**
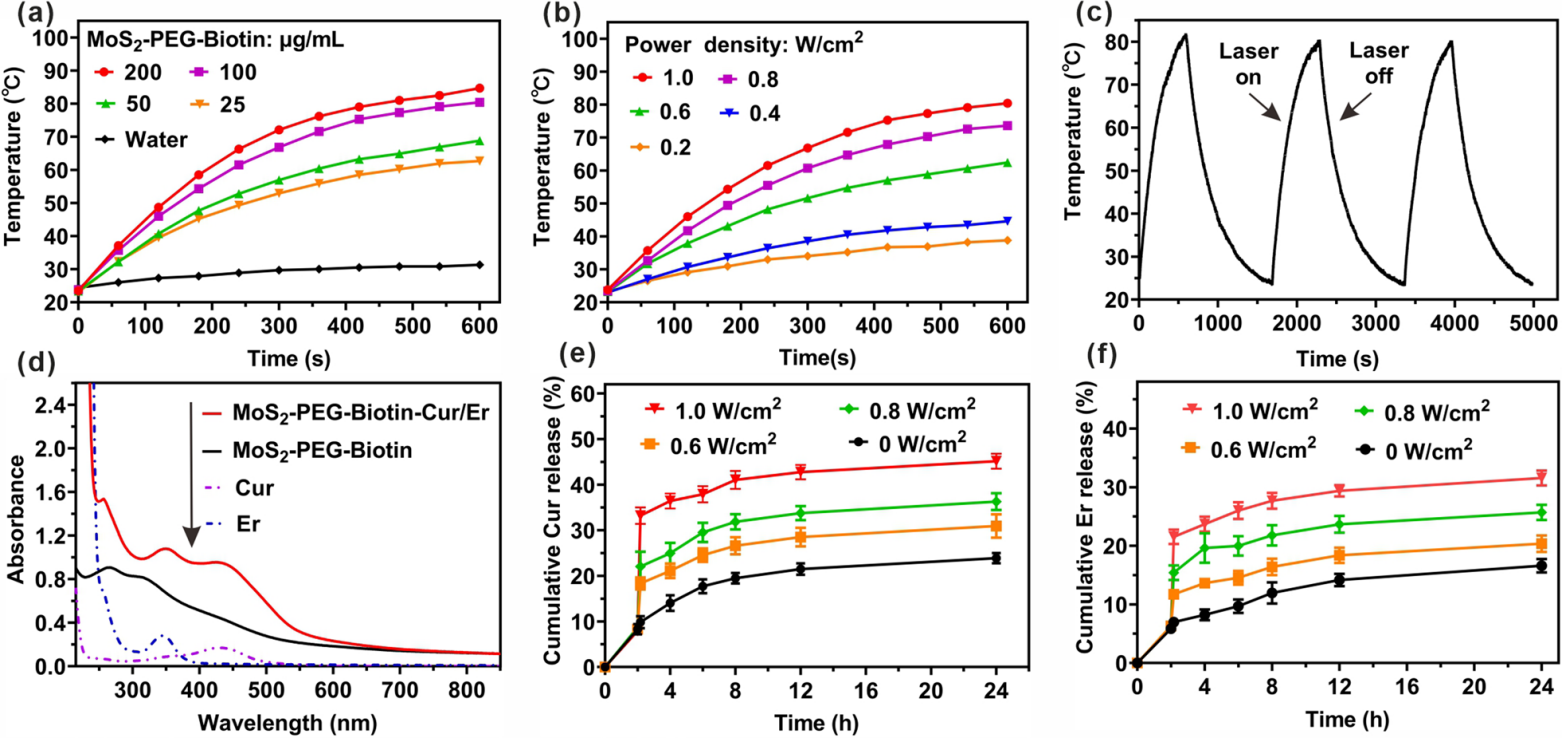
**a** Temporal temperature elevation of MoS_2_-PEG-Biotin at different concentrations upon NIR irradiation (1 W/cm^2^). **b** Temporal temperature elevation of MoS_2_-PEG-Biotin (100 μg/mL) during NIR irradiation at different power densities. **c** Temperature variation of MoS_2_-PEG-Biotin (100 μg/mL) over 3 cycles of NIR irradiation (1 W/cm^2^ for 600 s) and natural cooling. **d** UV–Vis–NIR spectra of MoS_2_-PEG-Biotin before and after Cur or Er loading. Release profiles of Cur (**e**) or Er (**f**) without and with NIR irradiation for 10 min

We evaluated the photothermal performance of MoS_2_-PEG-Biotin by monitoring the temperature variations of the nanosheets across a range of concentrations under NIR irradiation (1 W/cm^2^ for 600 s). MoS_2_-PEG-Biotin showed concentration-dependent temperature elevations, reaching a peak temperature increase (at 200 µg/mL) of up to 84.7 °C. In comparison, the temperature of water rose by a mere 4 °C (Fig. 3a). The photothermal transduction capacity was further explored by examining the temperature alterations of MoS_2_-PEG-Biotin (100 µg/mL) as a function of 808-nm laser power densities, revealing a distinct laser-power dependency (Fig. 3b). The photothermal stability of MoS_2_-PEG-Biotin was assessed through tracking temperature fluctuations during three cycles of laser irradiation on and off, which demonstrated consistent temperature changes and outstanding photothermal stability (Fig. 3c). These findings indicate that MoS_2_-PEG-Biotin possesses potent NIR absorption, remarkable photothermal conversion efficiency, and exceptional photothermal stability, rendering it a highly promising photothermal agent.

### Drug loading and NIR light-responsive release

Cur and Er were co-loaded onto MoS_2_-PEG-Biotin by first dissolving Cur in DMSO and combining it with the nanosheets, followed by the addition of Er. Undissolved or unbound drugs were removed through multiple centrifugation and ultrafiltration steps. The successful co-loading of Cur and Er onto MoS_2_-PEG-Biotin was verified by identifying two distinct peaks at 430 nm (characteristic of Cur) and 335 nm (characteristic of Er) in the UV–vis spectra of MoS_2_-PEG-Biotin-Cur/Er. The Cur and Er loading ratios (weight ratio of the drug compared to MoS_2_-PEG-Biotin) were determined to be approximately ~ 22.3% and ~ 10.1% for Cur and Er, respectively, under the experimental conditions (Fig. 3d).

Drug release behaviors were investigated through dialysis in PBS containing 0.5 wt% Tween-80, both with and without NIR light exposure. In the absence of stimulation, only ~ 7.4% of Cur was released within 2 h, whereas NIR irradiation (0.6 W/cm^2^) elicited a rapid surge in the cumulative release percentage of Cur by ~ 9.4% (from ~ 7.4% to ~ 16.8%) within 10 min (Fig. 3e). Without NIR irradiation, the subsequent 50 min saw only ~ 3.1% of Cur being released, suggesting that NIR light could initiate the release of Cur from MoS_2_-PEG-Biotin-Cur/Er through the accelerated motion of drug molecules driven by the heat generated by MoS_2_ during NIR irradiation [37]. Furthermore, the NIR light-triggered drug release exhibited a pronounced laser power-dependent increase, achieving the highest cumulative release percentage of Cur up to ~ 42.3% within 24 h. The release behavior of Er, both with and without NIR light, was consistent with that of Cur, demonstrating NIR light-responsive drug release (Fig. 3f). Such NIR light-triggered drug release is anticipated to enhance the chemotherapeutic efficacy of MoS_2_-PEG-Biotin-Cur/Er.

### Biotin-targeting-enhanced cellular uptake

**Fig. 4:**
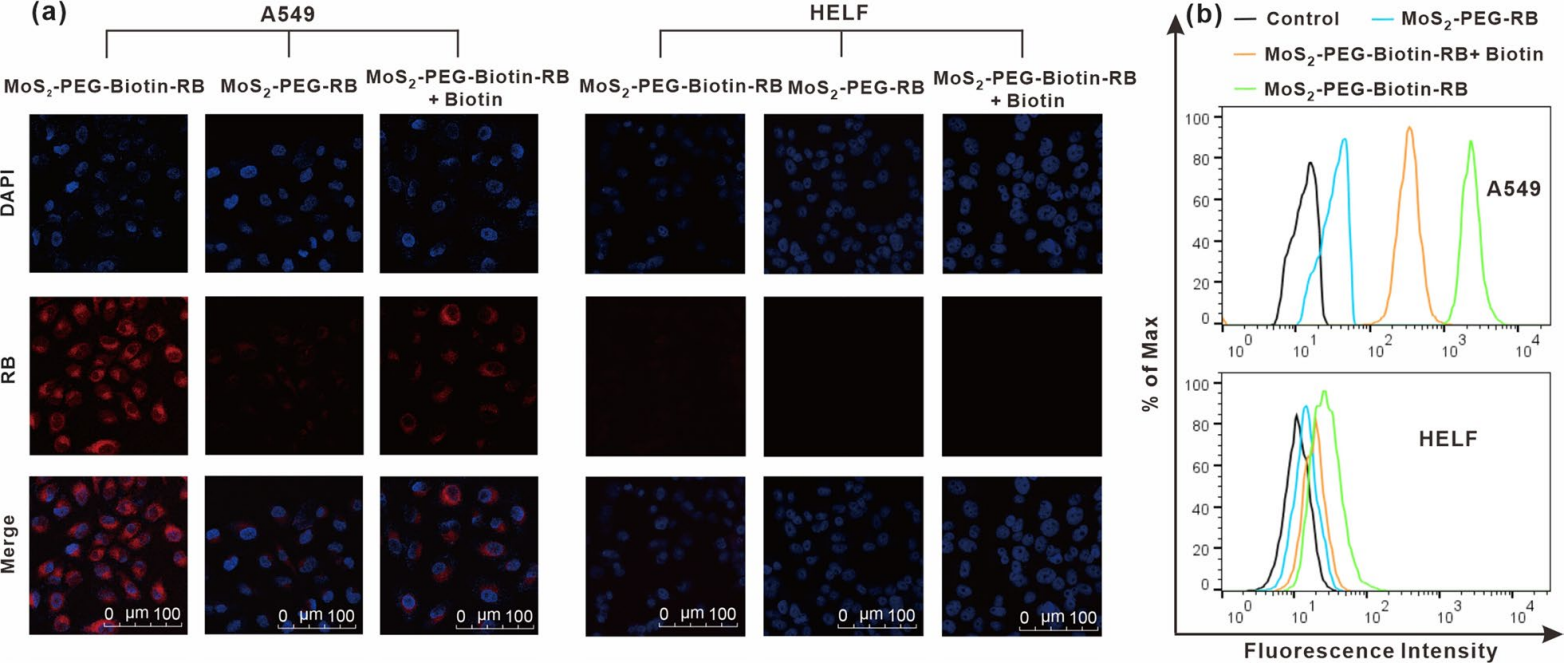
**a** Confocal images of A549 cells after various treatments, with HELF cells as a control. In this experiment, A549 and HELF cells were incubated with fluorescence labeled-MoS_2_ nanosheets (MoS_2_-PEG-Biotin-RB or MoS_2_-PEG-RB) for 2 h, washed with PBS, and stained with cell nucleus dye prior to the confocal microscopy observation. **b** Flow cytometry analysis of the intracellular RB fluorescence intensity in (**a**)

To explore the cellular uptake of biotin-modified MoS_2_ nanosheets, we synthesized a fluorescent probe (MoS_2_-PEG-Biotin-RB) by attaching a fluorescent dye RB to MoS_2_-PEG-Biotin (Additional file 1: Fig. S2). A549 and HELF cells were treated with MoS_2_-PEG-Biotin-RB or MoS_2_-PEG-RB for 2 h, washed twice with PBS, and stained with DAPI before visualization with a laser scanning confocal microscope. HELF cells incubated with MoS_2_-PEG-Biotin-RB or MoS_2_-PEG-RB showed relatively weak RB fluorescence (Fig. 4a). On the other hand, A549 cells incubated with MoS_2_-PEG-Biotin-RB exhibited significantly stronger RB fluorescence compared to those treated with MoS_2_-PEG-RB, suggesting that the presence of biotin may facilitate the uptake of MoS_2_-based nanosheets by cancer cells. To confirm this speculation, A549 cells were pre-treated with free biotin, followed by an additional 2 h incubation with MoS_2_-PEG-Biotin-RB. As expected, a weaker fluorescence was observed in the biotin-pretreated A549 cells, indicating that the cellular uptake of MoS_2_-PEG-Biotin-RB was impeded by free biotin. The confocal images were validated by flow cytometric analysis (Fig. 4b), further substantiating the concept of biotin-mediated cancer cell targeting. Therefore, active targeting may potentially amplify the accumulation of Cur and Er loaded by the biotin-modified MoS_2_ nanosheets in tumor tissues.

### Cytotoxicity and apoptosis in vitro

**Fig. 5:**
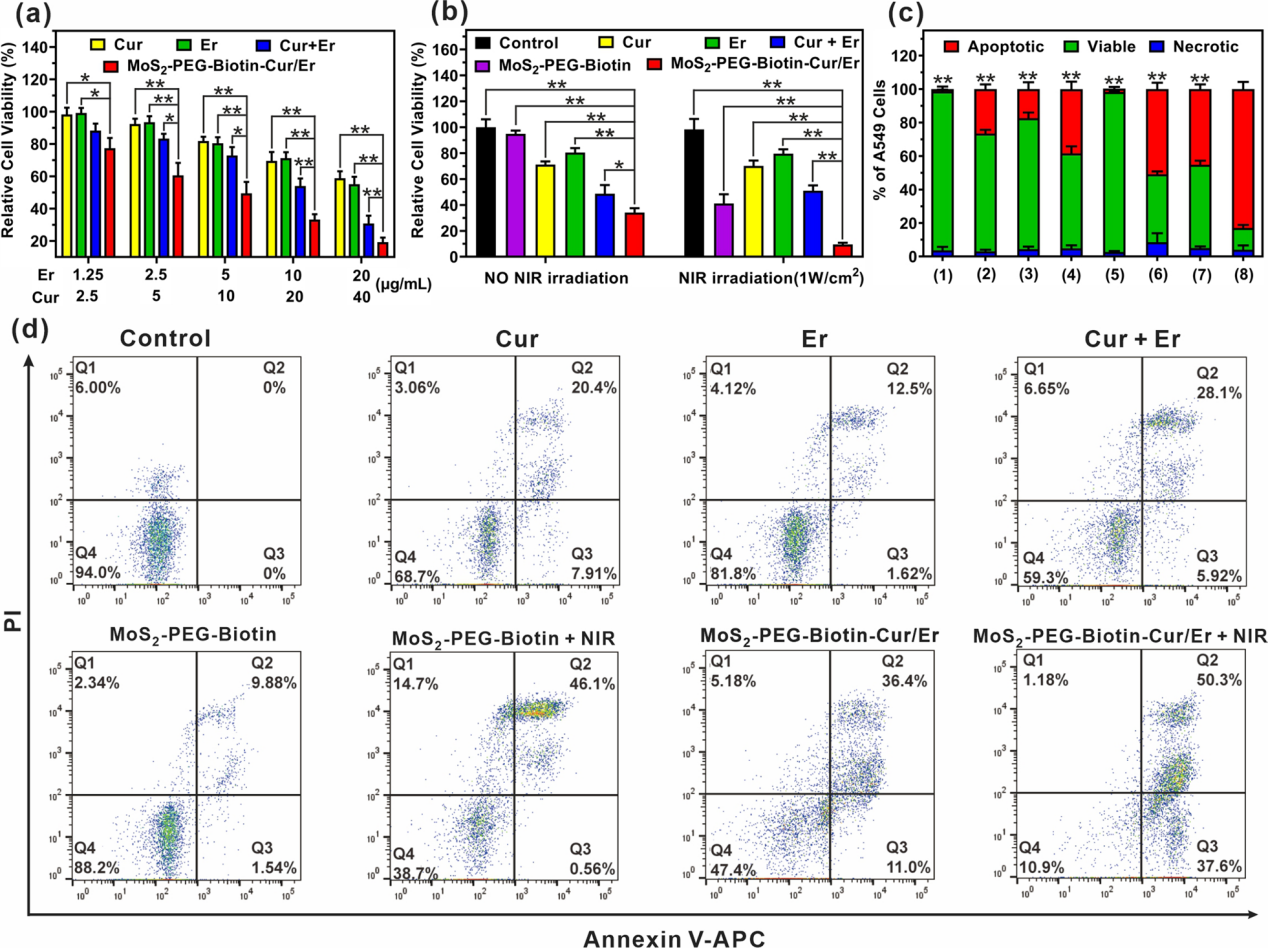
**a** The relative cell viability of A549 cells treated with Cur, Er, Cur + Er, and MoS_2_-PEG-Biotin-Cur/Er as a function of Cur or Er concentration. Significant differences between the MoS_2_-PEG-Biotin-Cur/Er group and the other groups are indicated as *p < 0.05 and **p < 0.01. **b** The relative cell viability of A549 cells after various treatments. The cells were treated with Cur, Er, Cur + Er, MoS_2_-PEG-Biotin, and MoS_2_-PEG-Biotin-Cur/Er ([MoS_2_-PEG-Biotin] = 100 μg/mL, [Cur] = 20 μg/mL, [Er] = 10 μg/mL) for 2 h, washed with PBS, and treated with fresh cell medium. After exposure to NIR irradiation (0 or 1 W/cm.^2^) for 10 min, the cells were incubated for another 48 h before the MTT assay. Significant differences between the MoS_2_-PEG-Biotin-Cur/Er group and the other groups are indicated as *p < 0.05 and **p < 0.01. **c**, **d** Flow cytometry analysis of apoptotic A549 cells after various treatments mentioned in section “Cell apoptosis”. Significant differences between the MoS_2_-PEG-Biotin-Cur/Er + NIR group and other experimental groups are indicated as **p < 0.01

Encouraged by the biotin-mediated cancer cell targeting, we assessed the cytotoxicity of MoS_2_-PEG-Biotin-Cur/Er using the MTT assay. Both a single drug and the combination of Cur and Er showed dose-dependent cytotoxicity, with the combination exhibiting higher cytotoxicity as a result of the synergistic chemotherapy (Fig. 5a). Co-loading of Cur and Er onto MoS_2_-PEG-Biotin further enhanced their cytotoxicity, facilitated by biotin-targeted enhanced cellular uptake.

We also investigated whether MoS_2_-PEG-Biotin-Cur/Er could induce greater cancer cell-killing effects after NIR irradiation owing to the excellent photothermal effect of MoS_2_-PEG-Biotin-Cur/Er with NIR light-triggered drug release. A549 cells were treated with Cur, Er, Cur + Er, MoS_2_-PEG-Biotin, and MoS_2_-PEG-Biotin-Cur/Er ([MoS_2_-PEG-Biotin] = 100 μg/mL, [Cur] = 20 μg/mL, and [Er] = 10 μg/mL), washed, and exposed to NIR irradiation (1W/cm^2^) for 10 min, followed by another 48 h of incubation prior to the MTT assay. Cur, Er, and Cur + Er groups showed no significant changes in cell viability with or without NIR irradiation (Fig. 5b). However, A549 cells treated with MoS_2_-PEG-Biotin exhibited a significant reduction in cell viability (from ~ 94.9 to ~ 41.4%) due to the heat generated upon NIR irradiation, damaging cancer cells. Moreover, MoS_2_-PEG-Biotin-Cur/Er demonstrated the most potent cell-killing effect with NIR irradiation due to the combination of synergistic chemotherapy and PTT inhibiting the proliferation of cancer cells. Annexin-V-FITC/PI assay confirmed the strongest cancer cell-killing effect, with MoS_2_-PEG-Biotin-Cur/Er inducing the highest apoptotic response (~ 89.2%) upon NIR irradiation, consistent with the MTT assay data (Fig. 5c and d).

### Tissue biodistribution of MoS_2_-PEG-Biotin-Cur/Er

**Fig. 6:**
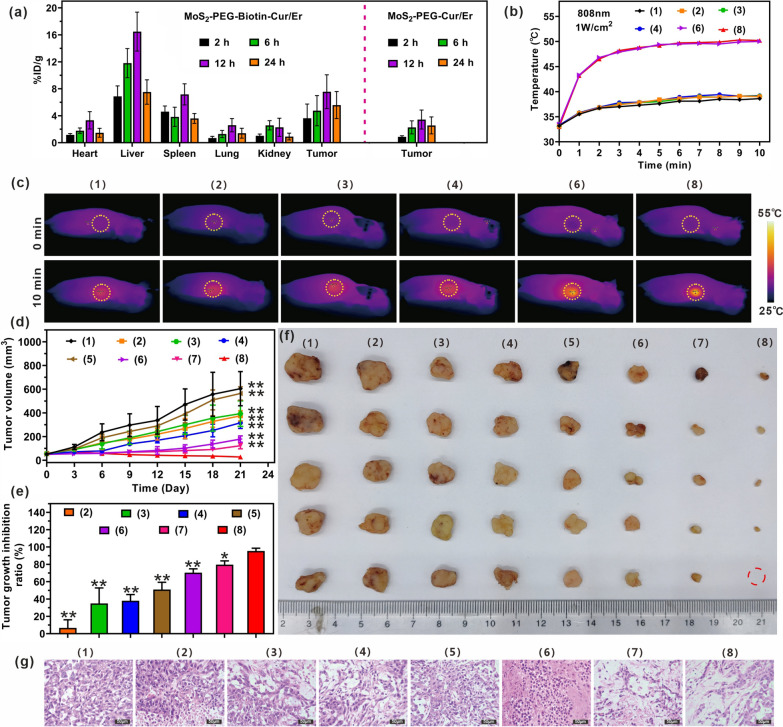
Mice were divided into eight groups as follows: (1) PBS as a control, (2) MoS_2_-PEG-Biotin, (3) Cur, (4) Er, (5) Cur + Er, (6) MoS_2_-PEG-Biotin + NIR, (7) MoS_2_-PEG-Biotin-Cur/Er, and (8) MoS_2_-PEG-Biotin-Cur/Er + NIR, **a** Tissue biodistribution of Mo^2+^ measured by ICP-MS after intravenous administration of MoS_2_-PEG-Biotin-Cur/Er and MoS_2_-PEG-Cur/Er as a function of time. **b** Temperature changes of tumors during NIR irradiation. **c** Thermal images of tumor-bearing mice in (**b**). **d** The tumor growth curve of all groups during 21-day treatments. Significant differences between the MoS_2_-PEG-Biotin-Cur/Er + NIR group and the other groups are indicated as **p < 0.01. **e** The tumor growth inhibition ratio of the experimental groups. Significant differences between the MoS_2_-PEG-Biotin-Cur/Er + NIR group and the other groups are indicated as **p < 0.01. **f** Photographs of tumors from each group after 21 days. **g** HE images of tumors from each group

The tissue biodistribution of MoS_2_-PEG-Biotin-Cur/Er was investigated by measuring the amount of Mo in major organs and tumors using ICP-MS. Following a 2 h intravenous injection, Mo rapidly disseminated to various organs, predominantly accumulating in the liver and spleen due to the mononuclear phagocytic system (Fig. 6a). The amount of Mo in major organs peaked at 12 h and declined at later time points due to the oxidization of MoS_2_-based nanosheets to water-soluble Mo (VI) oxide species, which could be excreted from the body through renal and fecal pathways. Utilizing the biotin-mediated tumor targeting strategy, the Mo levels in tumors of mice treated with MoS_2_-PEG-Biotin-Cur/Er were 4.15-, 2.08-, 2.20-, and 2.18-fold higher than those of MoS_2_-PEG-Cur/Er at 2, 6, 12, and 24 h post-i.v. injection, validating the role of biotin in facilitating the nanosheets' accumulation at the tumor site.

### In vivo synergistic chemotherapy and PTT

In this experiment, mice with a tumor volume of ~ 60 mm^3^ were divided into eight groups and treated with different agents. NIR irradiation was carried out after intravenous injection for 12 h, based on tissue biodistribution data, and temperature changes were recorded using a thermal camera. MoS_2_-based nanosheets showed a rapid increase in tumor temperature due to their biotin-mediated tumor-targeting capacity, resulting in hyperthermia in tumors (Fig. 6b and c). More importantly, the hyperthermia (> 48.0 °C) after 3 min of NIR irradiation remained almost unchanged during the subsequent 7 min of irradiation and could effectively induce the photothermal ablation of tumors [38].

During the 21-day treatments, tumor volumes were recorded every three days for all groups of mice. MoS_2_-PEG-Biotin alone showed low toxicity, with a rapid increase in tumor volume over time, reaching ~ 569.3 mm^3^ on day 21(Fig. 6d–f). Cur + Er exhibited stronger inhibition of tumor growth than Cur or Er alone in the first 6 days, but became uncontrollable during the subsequent 15 days due to the diminished effect of synergistic treatment caused by different pharmacokinetic processes. The co-loading of Cur and Er on MoS_2_-PEG-Biotin effectively delivered the two drugs to the tumor site through the biotin-mediated tumor-targeting ability, achieving a strong tumor growth inhibition ratio of ~ 70.4%, with ~ 179.3 mm^3^ on day 21. More importantly, MoS_2_-PEG-Biotin-Cur/Er achieved the highest tumor growth inhibition ratio of ~ 95.6% among these groups under NIR irradiation, with the lowest tumor volume growth of ~ 27.8 mm^3^, mainly due to the combination of enhanced synergistic chemotherapy and PTT. Histological analysis confirmed the excellent antitumor effect of MoS_2_-PEG-Biotin-Cur/Er, with most of the tumor cells from the MoS_2_-PEG-Biotin-Cur/Er + NIR group showing cell necrosis and lysis (Fig. 6g). The safety of MoS_2_-PEG-Biotin-Cur/Er in vivo was confirmed by body weight changes and HE staining of major organs, showing no acute side effects or organ damage at the tested dose (Additional file 1: Fig. S3).

## Conclusions

We successfully developed a biotin-modified MoS_2_-based nanosheet system, MoS_2_-PEG-Biotin-Cur/Er, for targeted co-delivery of Cur and Er. Biotin was used as a targeting ligand to modify the surface of MoS_2_ nanosheets, resulting in MoS_2_-PEG-Biotin which exhibited remarkable physiological stability, low toxicity, good biocompatibility, and tumor-targeting ability. Upon exposure to NIR irradiation, MoS_2_-PEG-Biotin efficiently converted absorbed light into heat, inducing photothermal ablation of cancer cells and triggering the release of the co-loaded drugs for enhanced synergistic chemotherapy. The combination of biotin-mediated cancer cell targeting, enhanced synergistic chemotherapy, and PTT led to effective suppression of cancer cell proliferation, induction of apoptosis in vitro, and inhibition of tumor growth in vivo. Hence, MoS_2_-PEG-Biotin-Cur/Er with excellent antitumor efficacy and safety in vivo, represents a promising therapeutic agent for cancer therapy.

### Supplementary Information

**Additional file 1: Figure S1.** Hydrodynamic sizes (**a**) and Zeta potentials (**b**) of MoS_2_ and MoS_2_-PEG-Biotin as determined by DLS. **Figure S2. a** UV–Vis–NIR spectra of MoS_2_-PEG-Biotin before and after RB loading. **b** Fluorescence spectra of free RB and MoS_2_-PEG-Biotin-RB at the same RB concentration (5 μg/mL, λex = 550 nm). The fluorescence of MoS_2_-PEG-Biotin-RB was weaker than that of free RB at the same RB concentration, due to the partial fluorescence quenching caused by fluorescence resonance energy transfer. **Figure S3.** Mice were divided into eight groups as follows: (1) PBS as a control, (2) MoS_2_-PEG-Biotin, (3) Cur, (4) Er, (5) Cur + Er, (6) MoS_2_-PEG-Biotin + NIR, (7) MoS_2_-PEG-Biotin-Cur/Er, and (8) MoS_2_-PEG-Biotin-Cur/Er + NIR. **a** Body weights of mice in each group as a function of time. **b** H&E images of major organs collected from mice after 21 days of intravenous administration of PBS and MoS_2_-PEG-Biotin-Cur/Er nanosheets.

## Data Availability

Data will be available on reasonable request.

## References

[1] Sung H, Ferlay J, Siegel RL, Laversanne M, Soerjomataram I, Jemal A, Bray F (2021). Global cancer statistics 2020: GLOBOCAN estimates of incidence and mortality worldwide for 36 cancers in 185 countries. CA Cancer J Clin.

[2] Zhang SB, Hong M, Sun XY, Huang DN, He DH, Chen YF, Yuan Y, Liu Y-Q (2022). Silybin has therapeutic efficacy against non-small cell lung cancer through targeting of skp2. Acta Mater Med.

[3] Harrison PT, Vyse S, Huang PH (2020). Rare epidermal growth factor receptor (EGFR) mutations in non-small cell lung cancer. Semin Cancer Biol.

[4] Hayashi H, Nadal E, Gray JE, Ardizzoni A, Caria N, Puri T, Grohe C (2022). Overall treatment strategy for patients with metastatic NSCLC with activating EGFR mutations. Clin Lung Cancer.

[5] Yu HA, Suzawa K, Jordan E, Zehir A, Ni A, Kim R, Kris MG, Hellmann MD, Li BT, Somwar R (2018). Concurrent alterations in EGFR-mutant lung cancers associated with resistance to EGFR kinase inhibitors and characterization of MTOR as a mediator of resistanceconcurrent alterations in EGFR-mutant lung cancers. Clin Cancer Res.

[6] Zhang KR, Zhang YF, Lei HM, Tang YB, Ma CS, Lv QM, Wang SY, Lu LM, Shen Y, Chen HZ (2021). Targeting AKR1B1 inhibits glutathione de novo synthesis to overcome acquired resistance to EGFR-targeted therapy in lung cancer. Sci Transl Med..

[7] Shafiee M, Mohamadzade E, ShahidSales S, Khakpouri S, Maftouh M, Alireza Parizadeh S, Mahdi Hasanian S, Avan A (2017). Current status and perspectives regarding the therapeutic potential of targeting EGFR pathway by curcumin in lung cancer. Curr Pharm Des.

[8] Li S, Liu Z, Zhu F, Fan X, Wu X, Zhao H, Jiang L (2014). Curcumin lowers erlotinib resistance in non-small cell lung carcinoma cells with mutated EGF receptor. Oncol Res.

[9] Dai X, Zhang J, Guo G, Cai Y, Cui R, Yin C, Liu W, Vinothkumar R, Zhang T, Liang G (2018). A mono-carbonyl analog of curcumin induces apoptosis in drug-resistant EGFR-mutant lung cancer through the generation of oxidative stress and mitochondrial dysfunction. Cancer Manag Res.

[10] Javadi S, Rostamizadeh K, Hejazi J, Parsa M, Fathi M (2018). Curcumin mediated down-regulation of αVβ3 integrin and up-regulation of pyruvate dehydrogenase kinase 4 (PDK4) in Erlotinib resistant SW480 colon cancer cells. Phytother Res.

[11] Hesari A, Rezaei M, Rezaei M, Dashtiahangar M, Fathi M, Rad JG, Momeni F, Avan A, Ghasemi F (2019). Effect of curcumin on glioblastoma cells. J Cell Physiol.

[12] Yamauchi Y, Izumi Y, Yamamoto J, Nomori H (2014). Coadministration of erlotinib and curcumin augmentatively reduces cell viability in lung cancer cells. Phytother Res.

[13] Ghafouri-Fard S, Poornajaf Y, Hussen BM, Avval ST, Taheri M, Mokhtari M (2023). Deciphering the role of Hippo pathway in lung cancer. Pathol-Res Pract.

[14] Wang Y, Cheng J, Zhao D, Liu Y, Luo T, Zhong YF, Mo F, Kong XY, Song J (2020). Designed DNA nanostructure grafted with erlotinib for non-small-cell lung cancer therapy. Nanoscale.

[15] Obeid MA, Alsaadi M, Aljabali AA (2023). Recent updates in curcumin delivery. J Liposome Res.

[16] Ruan Y, Xiong Y, Fang W, Yu Q, Mai Y, Cao Z, Wang K, Lei M, Xu J, Liu Y (2022). Highly sensitive Curcumin-conjugated nanotheranostic platform for detecting amyloid-beta plaques by magnetic resonance imaging and reversing cognitive deficits of Alzheimer's disease via NLRP3-inhibition. J Nanobiotechnology.

[17] Wang D, Zhou J, Fang W, Huang C, Chen Z, Fan M, Zhang M-R, Xiao Z, Hu K, Luo L (2022). A multifunctional nanotheranostic agent potentiates erlotinib to EGFR wild-type non-small cell lung cancer. Bioact Mater.

[18] Yin X, Tang CS, Zheng Y, Gao J, Wu J, Zhang H, Chhowalla M, Chen W, Wee AT (2021). Recent developments in 2D transition metal dichalcogenides: phase transition and applications of the (quasi-) metallic phases. Chem Soc Rev.

[19] Gupta A, Ghosh S, Thakur MK, Zhou J, Ostrikov KK, Jin D, Chattopadhyay S (2021). Up-conversion hybrid nanomaterials for light-and heat-driven applications. Prog Mater Sci.

[20] Sethulekshmi A, Saritha A, Joseph K, Aprem AS, Sisupal SB (2022). MoS_2_ based nanomaterials: advanced antibacterial agents for future. J Control Release.

[21] Shi J, Li J, Wang Y, Cheng J, Zhang CY (2020). Recent advances in MoS_2_-based photothermal therapy for cancer and infectious disease treatment. J Mater Chem B.

[22] Wang J, Sui L, Huang J, Miao L, Nie Y, Wang K, Yang Z, Huang Q, Gong X, Nan Y (2021). MoS_2_-based nanocomposites for cancer diagnosis and therapy. Bioact Mater.

[23] Dhas N, Kudarha R, Garkal A, Ghate V, Sharma S, Panzade P, Khot S, Chaudhari P, Singh A, Paryani M (2021). Molybdenum-based hetero-nanocomposites for cancer therapy, diagnosis and biosensing application: current advancement and future breakthroughs. J Control Release.

[24] Wang Y, Meng H-M, Li Z (2021). Near-infrared inorganic nanomaterial-based nanosystems for photothermal therapy. Nanoscale.

[25] Liu T, Wang C, Gu X, Gong H, Cheng L, Shi X, Feng L, Sun B, Liu Z (2014). Drug delivery with PEGylated MoS_2_ nano-sheets for combined photothermal and chemotherapy of cancer. Adv Mater.

[26] Liu J, Zheng J, Nie H, Zhang D, Cao D, Xing Z, Li B, Jia L (2019). Molybdenum disulfide-based hyaluronic acid-guided multifunctional theranostic nanoplatform for magnetic resonance imaging and synergetic chemo-photothermal therapy. J Colloid Interface Sci.

[27] Liu J, Li F, Zheng J, Li B, Zhang D, Jia L (2019). Redox/NIR dual-responsive MoS_2_ for synergetic chemo-photothermal therapy of cancer. J Nanobiotechnology.

[28] Liu J, Zheng J, Nie H, Chen H, Li B, Jia L (2020). Co-delivery of erlotinib and doxorubicin by MoS_2_ nanosheets for synergetic photothermal chemotherapy of cancer. Chem Eng J.

[29] Bolla PK, Gote V, Singh M, Patel M, Clark BA, Renukuntla J (2020). Lutein-loaded, biotin-decorated polymeric nanoparticles enhance lutein uptake in retinal cells. Pharmaceutics.

[30] Balan V, Dodi G, Mihai C, Serban A, Ursachi V (2021). Biotinylated chitosan macromolecule based nanosystems: a review from chemical design to biological targets. Int J Biol Macromol.

[31] Li H, Bruce G, Childerhouse N, Keegan G, Mantovani G, Stolnik S (2023). Biotin receptor-mediated intracellular delivery of synthetic polypeptide-protein complexes. J Control Release.

[32] Wang Z, Liu B, Sun Q, Feng L, He F, Yang P, Gai S, Quan Z, Lin J (2021). Upconverted metal–organic framework janus architecture for near-infrared and ultrasound co-enhanced high performance tumor therapy. ACS Nano.

[33] Song X, Wang R, Gao J, Han X, Jin J, Lv C, Yu F (2022). Construction of a biotin-targeting drug delivery system and its near-infrared theranostic fluorescent probe for real-time image-guided therapy of lung cancer. Chin Chem Lett.

[34] Liu L, Liu F, Liu D, Yuan W, Zhang M, Wei P, Yi T (2022). A smart theranostic prodrug system activated by reactive oxygen species for regional chemotherapy of metastatic cancer. Angew Chem.

[35] Chou SS, De M, Kim J, Byun S, Dykstra C, Yu J, Huang J, Dravid VP (2013). Ligand conjugation of chemically exfoliated MoS_2_. J Am Chem Soc.

[36] Liu T, Liu Z (2018). 2D MoS_2_ nanostructures for biomedical applications. Adv Healthc Mater.

[37] Wang S, Chen Y, Li X, Gao W, Zhang L, Liu J, Zheng Y, Chen H, Shi J (2015). Injectable 2D MoS_2_-integrated drug delivering implant for highly efficient NIR-triggered synergistic tumor hyperthermia. Adv Mater.

[38] Li R-T, Zhu Y-D, Li W-Y, Hou Y-K, Zou Y-M, Zhao Y-H, Zou Q, Zhang W-H, Chen J-X (2022). Synergistic photothermal-photodynamic-chemotherapy toward breast cancer based on a liposome-coated core–shell AuNS@ NMOFs nanocomposite encapsulated with gambogic acid. J Nanobiotechnology.

