# Supplementary material for: Targeted co-delivery of curcumin and erlotinib by MoS2 nanosheets for the combination of synergetic chemotherapy and photothermal therapy of lung cancer
Source: J Nanobiotechnology. 2023 Sep 16;21:333. doi: 10.1186/s12951-023-02099-4 (PMC10505307; doi:10.1186/s12951-023-02099-4)
Supplement: Supplementary file 1 — Additional file 1: Figure S1. Hydrodynamic sizes (a) and Zeta potentials (b) of MoS2 and MoS2-PEG-Biotin as determined by DLS. Figure S2. a UV–Vis–NIR spectra of MoS2-PEG-Biotin before and after RB loading. b Fluorescence spectra of free RB and MoS2-PEG-Biotin-RB at the same RB concentration (5 μg/mL, λex = 550 nm). The fluorescence of MoS2-PEG-Biotin-RB was weaker than that of free RB at the same RB concentration, due to the partial fluorescence quenching caused by fluorescence resonance energy transfer. Figure S3. Mice were divided into eight groups as follows: (1) PBS as a control, (2) MoS2-PEG-Biotin, (3) Cur, (4) Er, (5) Cur + Er, (6) MoS2-PEG-Biotin + NIR, (7) MoS2-PEG-Biotin-Cur/Er, and (8) MoS2-PEG-Biotin-Cur/Er + NIR. a Body weights of mice in each group as a function of time. b H&E images of major organs collected from mice after 21 days of intravenous administration of PBS and MoS2-PEG-Biotin-Cur/Er nanosheets. [file 12951_2023_2099_MOESM1_ESM.docx]

**Targeted co-delivery of** **curcumin and erlotinib by MoS_2_ nanosheets for the**

**combination of synergetic chemotherapy and photothermal therapy**

**of lung cancer**


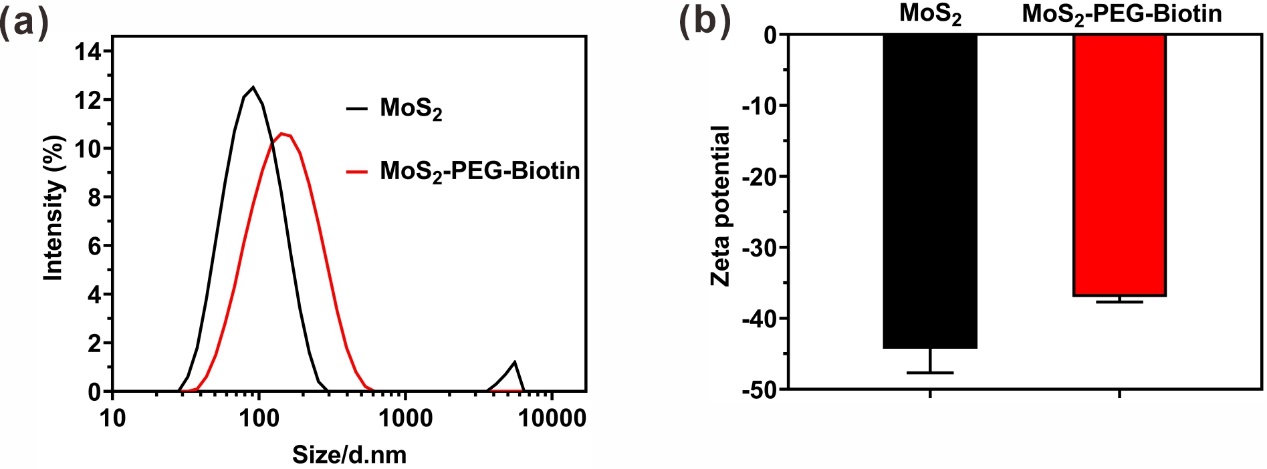


**Fig. S1.** Hydrodynamic sizes **(a)** and Zeta potentials **(b)** of MoS_2_ and MoS_2_-PEG-Biotin as determined by DLS.


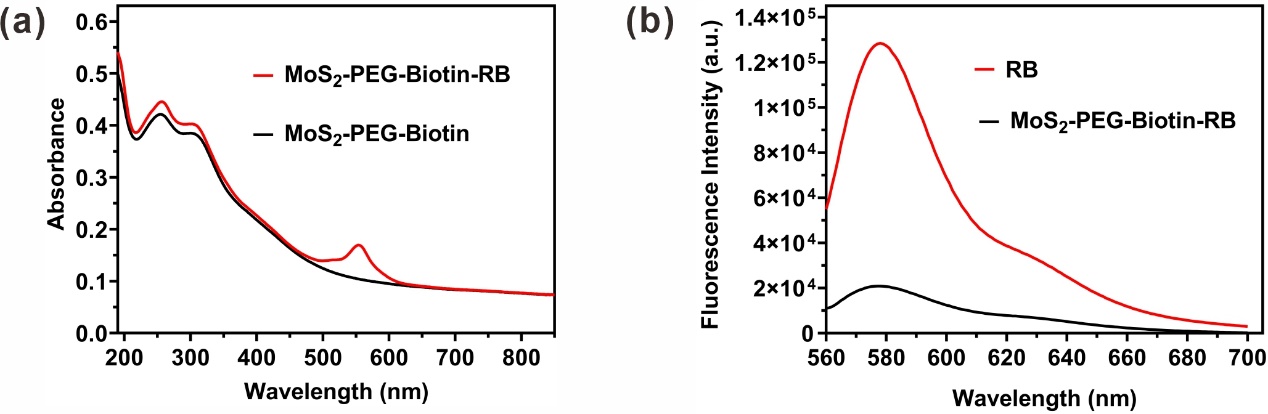


**Fig. S2. (a)** UV-vis-NIR spectra of MoS_2_-PEG-Biotin before and after RB loading. **(b)** Fluorescence spectra of free RB and MoS_2_-PEG-Biotin-RB at the same RB concentration (5 μg/mL, λex = 550 nm). The fluorescence of MoS_2_-PEG-Biotin-RB was weaker than that of free RB at the same RB concentration, due to the partial fluorescence quenching caused by fluorescence resonance energy transfer.


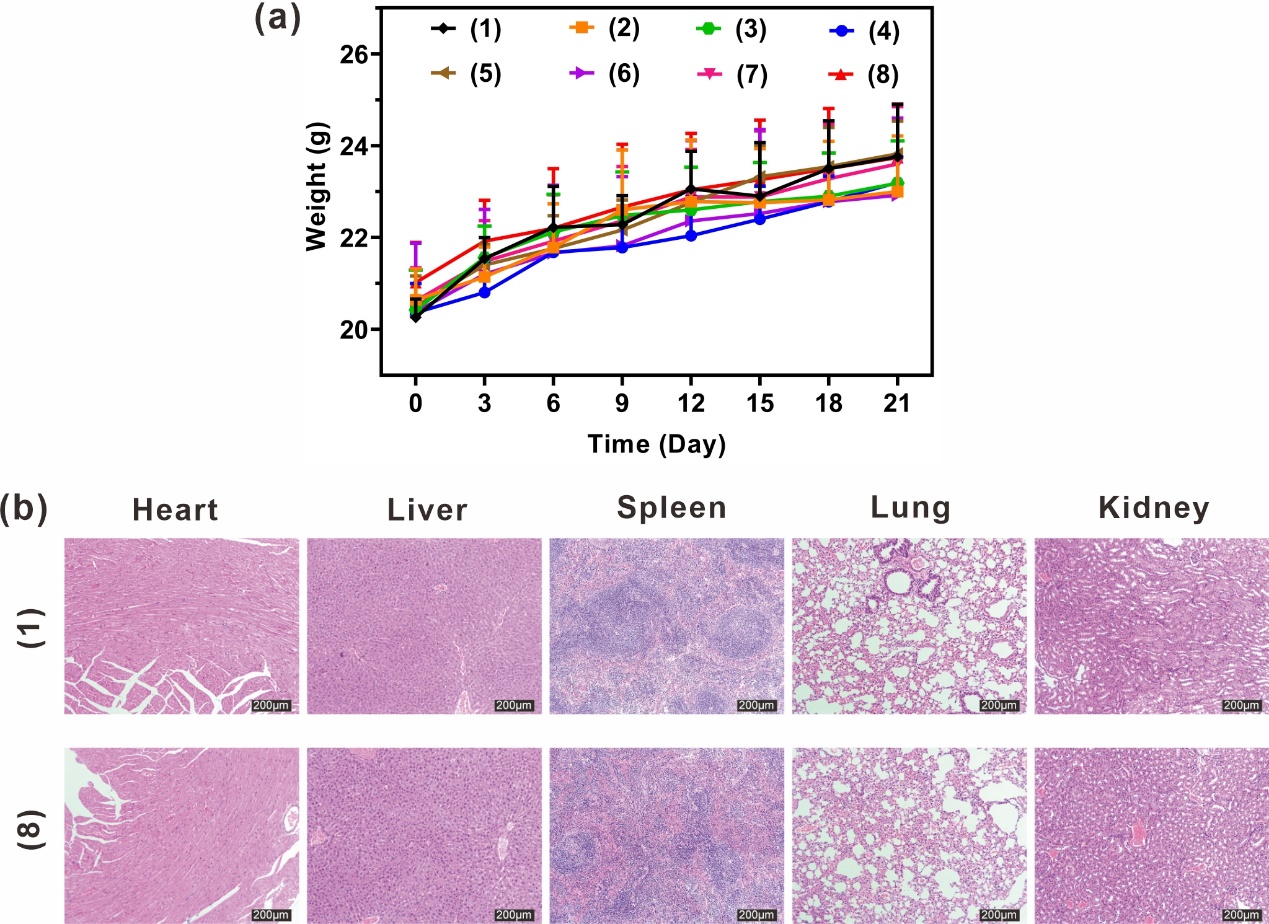


**Fig. S3.** Mice were divided into eight groups as follows: (1) PBS as a control, (2) MoS_2_-PEG-Biotin, (3) Cur, (4) Er, (5) Cur + Er, (6) MoS_2_-PEG-Biotin + NIR, (7) MoS_2_-PEG-Biotin-Cur/Er, and (8) MoS_2_-PEG-Biotin-Cur/Er + NIR. **(a)** Body weights of mice in each group as a function of time. **(b)** H&E images of major organs collected from mice after 21days of intravenous administration of PBS and MoS_2_-PEG-Biotin-Cur/Er nanosheets.
